# Immunoassay Methods and their Applications in Pharmaceutical Analysis: Basic Methodology and Recent Advances

**Published:** 2006-09

**Authors:** Ibrahim A. Darwish

**Affiliations:** *Department of Pharmaceutical Analytical Chemistry, Faculty of Pharmacy, Assiut University, Egypt*

**Keywords:** immunoassay, pharmaceutical analysis, drug discovery, pharmaceutical industry, antibodies

## Abstract

Immunoassays are bioanalytical methods in which the quantitation of the analyte depends on the reaction of an antigen (analyte) and an antibody. Immunoassays have been widely used in many important areas of pharmaceutical analysis such as diagnosis of diseases, therapeutic drug monitoring, clinical pharmacokinetic and bioequivalence studies in drug discovery and pharmaceutical industries. The importance and widespread of immunoassay methods in pharmaceutical analysis are attributed to their inherent specificity, high-throughput, and high sensitivity for the analysis of wide range of analytes in biological samples. Recently, marked improvements were achieved in the field of immunoassay development for the purposes of pharmaceutical analysis. These improvements involved the preparation of the unique immunoanalytical reagents, analysis of new categories of compounds, methodology, and instrumentation. The basic methodologies and recent advances in immunoassay methods applied in different fields of pharmaceutical analysis have been reviewed.

## INTRODUCTION

Immunoassays are bioanalytical methods in which the quantitation of the analyte depends on the reaction of an antigen (analyte) and an antibody. Principally, these methods are based on a competitive binding reaction between a fixed amount of labelled form of an analyte and a variable amount of unlabelled sample analyte for a limited amount of binding sites on a highly specific anti-analyte antibody. When these immunoanalytical reagents are mixed and incubated, the analyte is bound to the antibody forming an immune complex. This complex is separated from the unbound reagent fraction by physical or chemical separation technique. Analysis is achieved by measuring the label activity (e.g. radiation, fluorescence, or enzyme) in either of the bound or free fraction. A standard curve, which represents the measured signal as a function of the concentration of the unlabelled analyte in the sample is constructed. Unknown analyte concentration is determined from this calibration curve (1).

Immunoassay methods have been widely used in many important areas of pharmaceutical analysis such as diagnosis of diseases, therapeutic drug monitoring, clinical pharmacokinetic and bioequivalence studies in drug discovery and pharmaceutical industries (2). The analysis in these areas usually involves measurement of very low concentrations of low molecular weight drugs (3-6), macromolecular biomolecules of pharmaceutical interest (7), metabolites (8), and/or biomarkers which indicate disease diagnosis (9-13) or prognosis (14). The importance and widespread of immunoassay methods in pharmaceutical analysis are attributed to their inherent specificity, high-throughput, and high sensitivity for the analysis of wide range of analytes in biological samples. The detection system in immunoassays depends on readily detectable labels (e.g. radioisotopes or enzymes) coupled to one of the immunoanalytical reagents (i.e. analyte or antibody). The use of these labels in immunoassays results in assay methods with extremely high sensitivity and low limits of detection (15, 16). In circumstances whereas the specific measurements of large molecules at the femtomole to attomole level in complex biological matrices is required, no doubt that immunoassays are the methods of choice because of their high specificity and sensitivity (17-19).

In the early stages of drug discovery and development, particularly during the clinical pharmacokinetic studies for the new drug candidate, screening of large number of samples is required. This can be achieved only by using an analytical method of high throughput (20-22). The analysis of complex biological matrices (e.g. blood or urine) by immunoassay methods, being based on a specific binding reaction, can be achieved without pretreatment for the sample (23-25). Although the developing of a new immunoassay method for an analyte may take months (due to the time needed for generating the desired antibody), however, once suitable immuoanalytical reagents become available, the immunoassay method can be established in a time frame that is competitive with chromatographic methods. Furthermore, novel techniques were developed to enable the rapid production of specific antibodies. These techniques resulted in dramatic shortening of the time required for developing of immunoassay methods (26, 27). These potential advantages of immunoassay methods, in addition to the relatively low cost of the instruments, tools, or the reagents made immunoassays the methods of choice in many areas of pharmaceutical analysis.

## REAGENTSTS REQUIRED FOR IMMUNOASSAY DEVELOPMENT

These reagents are the antibodies, signal-generating labels, and separation matrices. Antibodies are the key reagents on which the success of any immunoassay depends. The antibodies can be either polyclonal or monoclonal. However, for immunoassay development for pharmaceutical analysis purposes, monoclonal antibodies are more advantageous than polyclonal ones (28-31). This is attributed to their higher degree of affinity and specificity towards the analyte. Even that, many successful immunoassays were developed using polyclonal antibodies because it was possible to generate the antibodies with high affinity to the analyte (32-37).

The signal generating labels in immunoassays include radioactive atoms (mostly 125I, 3H, and 14C) (38, 39). The use of radioactive labels offers extremely sensitive and quite precise assays, however, they have some drawbacks (e.g. health hazards, special attention for handling of the reagents, training of staff, short half-life time of the isotope, and expensive instrumentation for the counting of radioactivity. Therefore, alternative non-radioactive labels such as enzymes (40-49), fluorescent probes (50), chemiluminescent substances (51-53), metals and metal chelates (54-57), and liposomes (58) were introduced. On the basis of number of publications, enzymes are the most common labels employed in immunoassay methods for pharmaceutical compounds. A potential advantage in the use of enzyme labels for immunoassay is the possibility of the amplification of the signal, and subsequently the potential increasing in the sensitivity of the method. This is beneficial when the original signal is not sufficient to get the desirable sensitivity for the analysis.

The matrices used for separation of the immune complexes that formed as a result of immunoanalytical reactions include charcoal (59), polyethylene glycol (60), second antibody (61), microbeads (62, 63) or the most useful 96-well microwell plates; each well of the plate serves as a separate reaction tube. One component of the reaction (analyte or antibody) is coated onto the surface of the bottom of the plate wells, and the immune complex is formed on the surface of the wells. The use of these plates facilitates the washing steps, and reagents pipetting, and thus leads to semi-automation of the method (64, 65).

## BASIC METHODOLOGY INVOLVED IN PHARMACEUTICAL ANALYSIS

**Figure 1:**
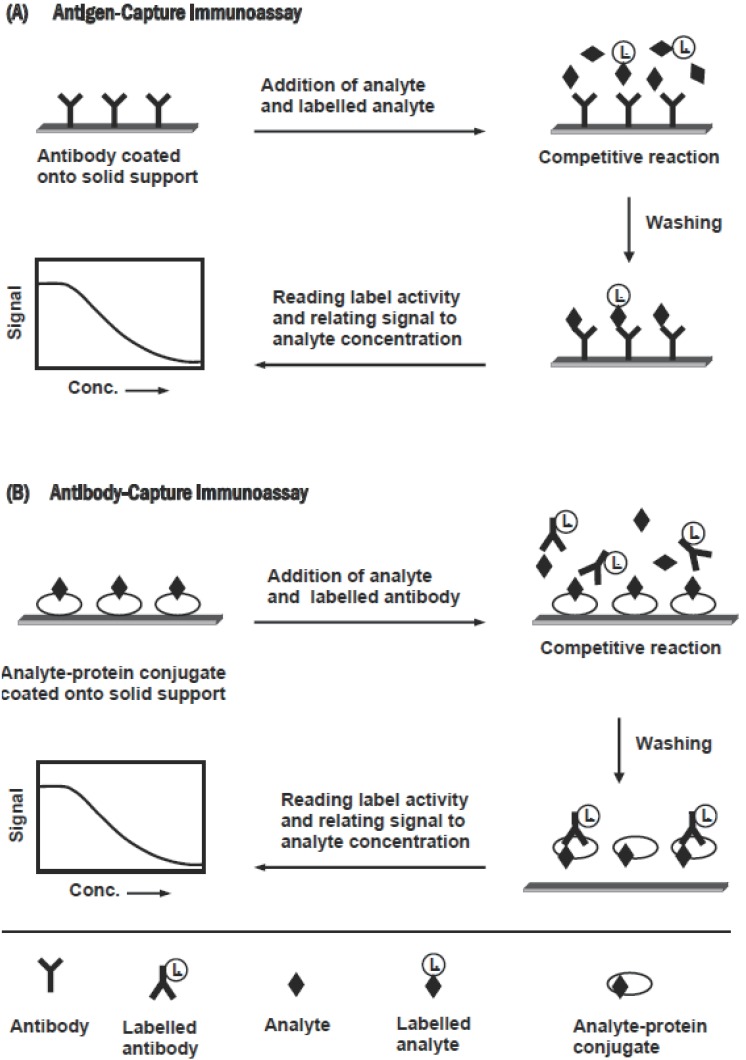
Schematic diagram for the competitive immunoassays.

**Figure 2:**
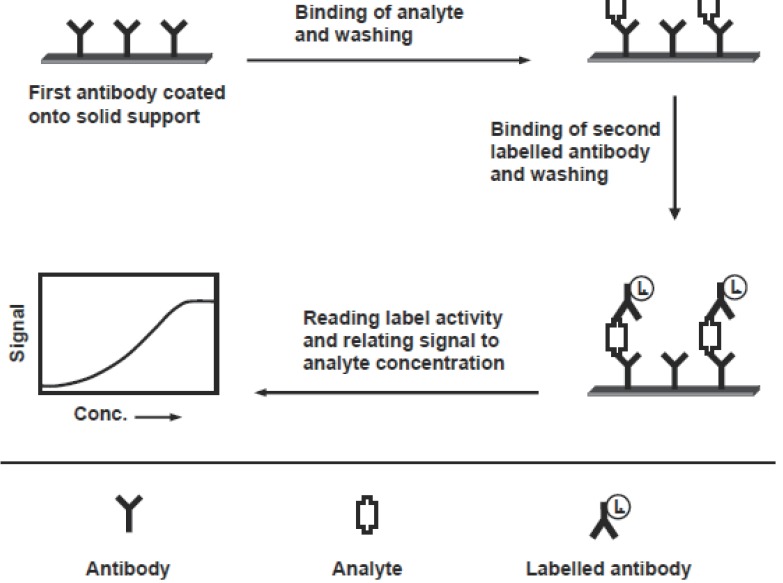
Schematic diagram for the non-competitive immunoassay.

Immunoassay methods that have been applied in pharmaceutical analysis, based on whether the separation step is or is not required, can be classified into heterogeneous or homogeneous assay, respectively. These methods can be performed in either competitive or non-competitive designs. The choice from these designs is based on nature of the analyte, labeling chemistry available and the analytical parameter required from the assay (e.g. sensitivity, dynamic range, and precision). Competitive design of immunoassays can be carried out in an antigen-capture or antibody-capture format, depending on whether the solid phase is coated with antibody or antigen (analyte), respectively. The features of these formats are shown in Figure 1. In the antigen-capture format (Figure 1A), the competition reaction occurs between the analyte (in sample) and a labelled analyte for the binding to a limited amount of anti-analyte antibody coated onto a solid support. After equilibration and separation, the label activity on the solid phase is measured, and the measured signal is inversely correlated to the concentrations of analyte in the sample (66). In antibody-capture format (Figure 1B), the analyte (or its protein conjugate) is coated onto a solid support. The competition occurs between the analyte (in sample) and the immobilized analyte for the binding to a limited amount of labelled anti-analyte antibody. After equilibration and separation, the activity of the label bound to the solid support is measured, and the signal is inversely correlated to the concentration of the analyte. The non-competitive design (usually called “two-site” or “sandwich” assay) is used for large analytes possessing more than one recognition epitopes on the molecule. It requires two antibodies that bind to non-overlapping epitopes on the analyte molecules. One of the two antibodies is bound to the solid phase, and the second one is labelled and used for detection. Figure 2 illustrates the features of this assay. The sample analyte is allowed to bind to an immobilized antibody. After washing, the solid support (contains the formed analyte-antibody complex) is incubated with an excess of the second labelled antibody, which binds to the remaining epitope on the analyte molecule. After washing, the activity of the label bound to the solid support is measured.

## IMMUNOASSAY METHODS APPLIED IN PHARMACEUTICAL ANALYSIS

### Radioimmunoassay

The history and development of radioimmunoassay (RIA) were reviewed by Najjar and Weintraub (67). RIA methods have been used successfully for the determination of limitless number of pharmaceutically important compounds in biological fluids. Most of these methods are now automated with separation assisted by the use of antibody bound to a solid phase matrices (57, 68-70). Table 1 shows list of the compounds that have been analyzed by RIA in biological fluids (71-97). The most important advantage of RIA in the measurement of compounds in biological fluids is the quite precision and extreme sensitivity, which can not be achieved by other analytical techniques (except GC-MS).

**Table 1:** List of Compounds Analyzed in Biological Fluids by Radioimmunoassay

Compound	Sample	Sensitivity	Ref.

Digoxin	Serum	0.52 ng/ml	(71)
Digitoxin	Serum	5 ng/ml	(72)
Oubain	Plasma	5 pmol	(73)
Tetracycline	Serum, urine	1 ng/ml	(74)
Isepamicin	Plasma	0.1 μg/ml	(75)
Acyclovir	Plasma	7 ng/ml	(76)
Tipredane	Plasma	1 ng/ml	(77)
Zopiclone	Urine	10 pg/ml	(78)
Imidapril	Plasma, urine	0.1 ng/ml	(79)
Hydroxyfluphenazine	Plasma	0.1 ng/ml	(80)
Benzotropine	Plasma	0.16 ng/ml	(81)
Trihexyphenidyl	Plasma	39 pg/ml	(82)
Zidovudine	Dried blood spot	24 pg/ml	(83)
Zolpidem	Serum, urine	0.1 ng/ml	(84)
Morphine	Urine	7.9 ng/ml	(85)
Cocaine	Hair, urine	0.1 ng/ml	(86)
Methadone	Hair	0.1 ng/ml	(87)
Thyroxine	Hair	31.47 pg/mg	(88)
Insulin	Serum	11 pmol	(89)
Estradiol	Saliva	0.25 pg/ml	(90)
Ethinylestradiol	Plasma	43 pg/ml	(56)
Estrone sulphate	Plasma	1.21 nmol	(91)
Androstenedione	Serum	0.5 nmol	(92)
Progesterone	Saliva	48 pmol	(93)
Pregnenolone	Plasma	0.15 nmol	(94)
1,25-Dihydroxy vitamin D	Plasma	0.2 pg/ml	(95)
Angiotensin II	Plasma	2.3 pg/ml	(96)
Prostaglandin	Plasma	100 pg/ml	(97)

### Enzyme Immunoassay

Enzyme immunoassay (EIA) is analogous to RIA except that the label is an enzyme rather than a radioisotope. The basic approach for use of an enzyme as an immunoassay label is appreciated by coupling an enzyme molecule into one of the immunoanalytical reagents (analyte or antibody), by appropriate chemical technique, and then carrying out the immunoanalytical reaction in the normal way. Following the separation of bound and free fractions, the enzyme activity is monitored in either of the two fractions. This is achieved by adding substrate, and subsequent monitoring the turnover of the substrate to product. The product must possess measurable physical or chemical differences from the substrate. For example, colourless chromogenic substrates which are converted into coloured products by the action of the enzyme label. The coloured products can be simply measured by a spectrophotometer. The measured signal is then correlated to the analyte concentration. Although enzyme/chromogenic substrate system is frequently used, however, many other systems can be used (40-48). A list for compounds that have been analyzed by competitive assays; antigen-capture (89-108) and antibody-capture (109-118) EIA are given in Tables 2 and 3, respectively. As well, sensitive non-competitive EIA methods were developed for various compounds of pharmaceutical interest (119-124); these methods are shown in Table 4. Homogenous EIA, exemplified by the enzyme multiplied immunoassay (EMIA) (49) is the major breakthrough in immunoassay technology. Homogenous EIA was successfully applied to therapeutic drug monitoring of some pharmaceutical compounds, e.g. aminoglycoside antibiotics (125). Reagent kits are available for the analysis of amikacin, gentamicin, netilmicin and tobramycin. These kits are used with the Viva automated analyser or with automated analysers from different manufacturers (54). Other homogenous EMIA were developed for measuring the concentration of thyroxine (126), triiodothyronine (127), theophylline (128), and cyclosporine (129) in biological fluids (Table 4).

**Table 2:** Antigen-Capture Enzyme Immunoassays for some Pharmaceutical Compounds

Compound	Sample	Enzyme	Substrate	Detection	Sensitivity	Ref.

Digoxin	Serum	β-GAL	DMAG	Photometric	0.2 ng/ml	(98)
Methamphetamine	Blood, Urine	HRP	TMB	Photometric	2 ng/ml	(99)
Benzodiazepine, morphine	Blood, urine	HRP	TMB	Photometric	5, 1 ng/ml	(99)
Phencyclidine, cannabinoids	Blood, urine	HRP	TMB	Photometric	25, 1 ng/ml	(99)
Metanephrine, normetanephrine	Urine	ALP	PNPP	Photometric	0.3 μmol	(100)
Zonisamide	Serum	β-GAL	NPGP	Photometric	μg/ml	(101)
Clenbuterol	Hair	ALP	PNPP	Photometric	12 pg/well	(102)
Dihydrostreptomycin	Milk	HRP	TMB	Photometric	0.01 μg/ml	(103)
Neomycin	Milk	HRP	TMB	Photometric	0.01 μg/ml	(103)
25-Hydroxy vitamin D3	Serum	HRP	TMB	Photometric	4.4 ng/ml	(104)
Medroxyprogesterone	Serum	ALP	ADP	Chemiluminescence	0.83 pg/well	(105)
17-β-Estradiol	Serum	HRP	TMB	Electrochemical	20 pg/ml	(106)
Oxytocin	Serum	ACE	ACI/DTNBA	Photometric	1.5 pg/ml	(107)
Osteocalcin	Serum	HRP	TMB	Photometric	4.45 ng/ml	(108)

β-GAL, β-D-galactosidase; HRP, horseradish peroxidase; ALP, alkaline phosphatase; ACE, acetylcholinesterase; DMAG, dimethylacridinone galactoside; TMB,3,3’,5,5’-tetramethylbenzidine; PNPP, p-nitrophenyl phosphate; NPGP, nitrophenylgalacto-pyranoside; ADP, adamantyl-1,2-dioxetane; ACI/DTNBA, acetylcholine iodide/5,5’-ditthiobis (2-nitrobenzoic acid).

**Table 3:** Antibody-Capture Enzyme Immunoassays for some Pharmaceutical Compounds

Analyte	Sample	Enzyme	Substrate	Detection	Sensitivity	Ref.

Streptomycin	milk	ALP	PNPP	Photometric	1.6 ng/ml	(109)
Spectinomycin	Kidney extract	ALP	PNPP	Photometric	0.5 ng/ml	(110)
Spectinomycin	Plasma	β-GAL	MUG	Photometric	2 ng/ml	(111)
Gentamicin	Serum	HRP	OPD	Fluorimetric	0.5 ng/ml	(112)
Oxacillin	milk	GOD	Glucose	Photometric	10 ng/ml	(113)
Dicloxacillin	milk	HRP	TMB	Photometric	30 ng/ml	(113)
Scopolamine	Plant extract, serum	ALP	PNPP	Photometric	0.1, 0.5 ng/ml	(114)
Ractopamine	Urine	HRP	TMB	Photometric	2.6 ng/ml	(115)
Fenoterol	Urine	HRP	TMB	Photometric	1.3 ng/ml	(115)
Dexamethasone	Urine	ALP	BCIP/NBT	Photometric	0.4 pg/ml	(116)
Digoxin	Serum	β-GAL	GPDA	Photometric	0.5 ng/ml	(117)
Oxazepam	Urine	ALP	PNPP	Photometric	0.3 μg/ml	(118)

ALP, alkaline phosphatase; β-GAL, β-D-galactosidase; HRP, horseradish peroxidase; GPb, glycogen phosphorylase b; G6PDH, glucose-6-phosphate dehydrogenase; PNPP, p-nitrophenylphosphate; NADPH, nicotinamide anednine dinucleotide phosphate; MUG, 4-methylumbelliferyl-β-D-galactose; TMB, 3,3’,5,5’-tetramethylbenzidine; SAV, streptavidin; NAD, nicotinamide adenine dinucleotide; G6P, glucose-6-phosphate.

**Table 4:** Non-Competitive Heterogeneous and Homogeneous Enzyme Immunoassay Methods for some Compounds of Pharmaceutical Interest

Compound	Sample	Enzyme	Substrate>	Detection	Sensitivity	Ref.

Non-Competitive Assays						
Tobramycin	Standard solution	ALP	PNPP	Photometric	0.05 μg/ml	(119)
Epinephrine, norepinephrine	Plasma	ALP	NADPH	Photometric	10; 20 pg/ml	(120)
Parathyroid hormone	Plasma	β-GAL	MUG	Fluorimetric	1 pg/ml	(121)
Thyroid stimulating hormone	Serum	HRP	TMB	Photometric	0.1 μIU	(122)
Insulin	Serum	ALP	SAV	Chemiluminescence	5 pmole	(123)
Low density lipoprotein	Blood	HRP	TMB	Photometric	1 U/ml	(124)
Homogenous Assays						
Thyroxine	Serum	GPb	Glycogen	Fluorimetric	10 ng/ml	(126)
triiodothyroxine	Serum	GPb	Glycogen	Fluorimetric	0.3 ng/ml	(127)
Theophylline	Serum	ALP	NAD	Electrochemical	6.3 μg/ml	(128)
Cyclosporins	Whole blood	G6PDH	G6P	Photometric	24.9 ng/ml	(129)

ALP, alkaline phosphatase; β-GAL, β-D-galactosidase; HRP, horseradish peroxidase; GPb, glycogen phosphorylase b; G6PDH, glucose-6-phosphate dehydrogenase; PNPP, p-nitrophenylphosphate; NADPH, nicotinamide anednine dinucleotide phosphate; MUG, 4-methylumbelliferyl-β-D-galactose; TMB, 3,3’,5,5’-tetramethylbenzidine; SAV, streptavidin; NAD, nicotinamide adenine dinucleotide; G6P, glucose-6-phosphate.

### Fluoroimmunoassay

Fluoroimmunoassay (FIA) is analogous to RIA except that the label is a fluorophore rather than a radioisotope. As in other immunoassays, FIA can be categorized into heterogeneous and homogeneous assays, depending on whether the separation step is or is not needed, respectively. Either of heterogeneous or homogeneous assays can be performed in a competitive or non-competitive format. Heterogeneous competitive FIA methods are currently available for analysis of various compounds of pharmaceutical importance in biological fluids: aminoglycoside antibiotics (130), morphine-3-glucuronide, the major urinary metabolite of heroin and morphine (131), benzoylecogonine in urine (132), and endocrine disruptTing chemicals (estrone, estradiol, and ethinylestradiol) in a complex synthetic aqueous matrix (133). These FIA offered sensitivities ranged from 0.01-2 ng/ml. A heterogeneous competitive FIA approach was developed for the measurement of the fluorescent signal directly on the solid supports, wells of microwell plates (134). This approach was used for the determination of the total thyroxine in human serum. In a further work, the fluoTrescence signal measured directly onto the solid phase was considerably stabilized by pretreatment of the wells with a glycerin solution. This treatment caused in an increase in the sensitivity of the assay (135). In homogeneous FIA, the antibody-bound analyte is not needed to be separated from the free analyte before the fluorescence measurement. Almost all the homogeneous FIA applied in pharmaceutical analysis are performed as competitive. In these assays, the antibody binding causes some changes in the fluorescence properties (e.g. polarization) of the labelled analyte. The analyte concentration in a sample can be monitored directly from the reaction mixture. The most common type of these assays is the fluorescence polarization fluoroimmunoassay (FPFIA). This technique is based on the following: when a fluorescent analyte conjugate is excited with polarized light, the polarization of the resulting emission depends inversely on the decay constant of the probe (4-5 ns for fluorescein isothiocyanate) and on the rotational motion of the conjugate. With small molecules (e.g. drugs), random rotation decreases the polarization signal; when bound to specific antibodies, their rotation slows, and the polarization signal increases. The increase in the polarization signal is related to the concentration of the analyte. Originally, this technique was developed for single tube analytical instruments, but the technology was rapidly converted Immunoassays into high-throughput screening assays when automated analyzers became commercially available (20, 57). The FPFIA methods utilizing automated analyzer are simple, precise, and easy to perform. Therefore, these methods are widely applied in drug discovery studies (21) as well as in therapeutic monitoring of a wide range of analytes (136-148), shown in Table 5. Recently, an improved one-step FPFIA was developed for analysis of progesterone hormone using an immunocomplex single reagent (a pre-equilibrated mixture of antibody and tracer) (149). This assay is a speedy and does not need incubation period before the measurement of the fluorescence polarization signal (the total assay time is about 7 min for 10 samples). The limit of detection of this assay was 2.7 ng/ml with 50 ml samples.

**Table 5:** Some Pharmaceutical Compounds Analyzed in Biological Fluids by Fluorescence Polarization Immunoassay

Compound	Sample	Ref.

tricyclic antidepressants		
Amitryptylene, clomipramine, clothiepin, doxepin	Urine	(136)
Antiepileptics		
Phenytoin, carbamazepine, phenobarbitone	Serum	(137)
Topiramate	Plasma	(138)
Antibiotics		
Amikacin, tobramycin	Serum	(139)
Vancomycin	Urine	(140)
Chemotherapeutics		
Cytosine arabinoside, nimustine	CSF^a^	(141)
Methotrexate	CSF	(142)
Stimulants for central nervous system		
Amphetamine, methamphetanmine	Urine	(143)
Pschycoactive drugs		
Nordizepam	Serum, urine	(144)
lorazepam	Serum, urine	(145)
Adinazolam	Serum	(146)
Methadone, benzoylecgonine	Blood	(147)
Cannabinoids	Urine	(148)

a Cerebrospinal fluid.

Time-resolved fluoroimmunoassay (TRFIA) is a separate group of FIA because its principles can be adapted to both heterogenous and homogenous assay format. In TRFIA, chelates of lanthanides and palladium ions are used as labels (54-57). Highly sensitive TRFIA methods were developed for analysis of many pharmaceutical compounds and hormones in biological fluids (Table 6). The advantage in these assays was the possibility to resolve the background fluorescence (due to biological fluids) from the assay fluorescence. Therefore, the non-specific endogenous fluorescence was eliminated.

**Table 6:** Some Pharmaceutical Compounds and Hormones Analyzed in Biological Fluids by Time-Resolved Fluoroimmunoassays

Compund	Sample	Sensitivity	Ref.

Chloropromazine	Serum	10 ng/ml	(55)
Desipramine	Serum	10 ng/ml	(55)
Methamphetamine	Serum	1 ng/ml	(55)
Zeranol	Urine	0.16 ng/ml	(150)
Thyroxine	Serum	1.07 pmol	(151)
Testosterone	Saliva	16 pmol	(152)
Estradiol fatty acid esters	Serum	36 pmol	(153)

### Chemiluminescence Immunoassay

Chemiluminescence immunoassay (CLIA) involves a chemiluminescent substance as a label. The growing success of this technique in pharmaceutical analysis due to its high performance, low detection limits, and good precision. The principles, methodology, instrumentation, and applications of chemiluminescence immunoassay are the subject of some books (38, 154) and reviews (52, 53, 57). The CLIA was used for analysis of many compounds of pharmaceutical importance (155-166); these compounds are shown in Table 7.

**Table 7:** List for Compounds of Pharmaceutical Importance Analyzed by Chemiluminescence Immunoassay

Compound	Sample	Sensitivity	Ref.

Antiepileptics			
Carbamazepine	Serum	0.13 μg/ml	(155)
phenobarbital	Serum	0.3 μg/ml	(155)
Phenytoin	Serum	0.45 μg/ml	(155)
Valproic acid	Serum	0.68 μg/ml	(155)
Bronchodilators			
Salbutamol, clenbuterol	Tissue	2 pg/well	(156)
Vitamins			
vitamin B12	Sea food	0.1 ng/ml	(157)
Hormones			
Insulin	Serum	2 μU/ml	(158)
Thyroxin	Serum	5.5 pmol	(159)
Estradiol	Saliva	3.8 pmol	(160)
Estrone	Serum	55 pmol	(161)
Testosterone	Serum	0.13 nmol	(162)
19-Nortestosterone	Urine	0.03 ng/ml	(163)
Medroxprogesterone acetate	Serum	0.83 pg/well	(164)
Phytoestrogens			
daidzein	Serum	10 pg/well	(165)
Tumor markers			
Prostat-specific antigen	Serum	1.7 pg/ml	(166)

### Liposome Immunoassay

**Figure 3:**
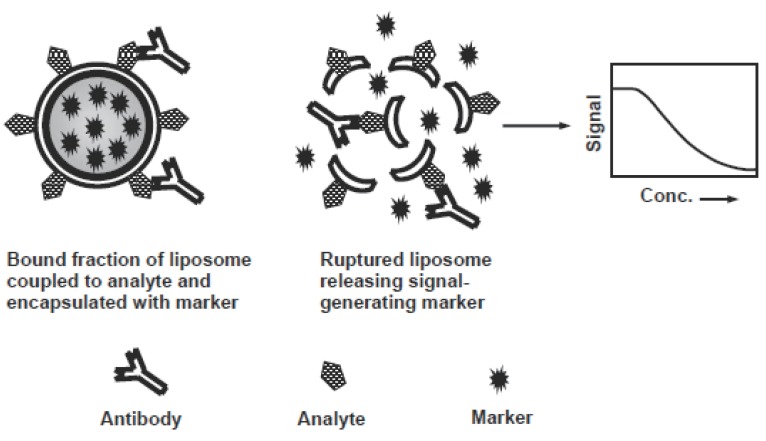
Schematic diagram for the liposome immunoassay.

Liposome immunoassay (LIA) is the assays involving a liposome-encapsulating marker. In LIA, liposomes are prepared and then coupled to either analyte or antibody by a suitable procedure (58), and then carrying out the assay in normal way. Detection in LIA relies on the lysis of the liposome and releasing the encapsulated marker, which is then measured and related to the analyte concentration. LIA methods are applied as homogenous assays often rely on the lytic activity of complement on antibody-associated liposomes, or the ability of free mellitin (a bee venom protein) conjugate with an analyte to lyse the liposomes, and release markers. Heterogeneous LIA are performed competitively or non-competitively in a similar way to stanTdard EIA, except replacement of the enzyme label with a liposome. Washing steps are used to separate bound liposome- labelled reagent from unbound reagents. All bound liposomes are lysed by addition of a detergent releasing the signal-generating marker (Figure 3). Different LIA methods (167-172) were developed for analysis of various pharmaceutical compounds (Table 8).

**Table 8:** Liposome Immunoassays for some Pharmaceutical Compounds

Compound	Liposome composition	Marker: (inside) outside	Lytic agent	Sample	Sensitivity	Ref.

Digoxin	PC-COL	FAD-GOD, CL reagent	Mellitin	Serum	10 pmol	(167)
Gentamycin	DMPC-COL	(Calcein)	Phospholipase C	Buffer	2.5 pg/ml	(168)
Biotin	lecithin, COL	(ALP) PNPP	Mellitin	Buffer	2 nmol	(169)
Carbamazepine	DMPC, DMPG, COL, PE	(PNPP) ALP	Guinea pig serum	Serum	4 ng/ml	(170)
Phenobarbital	DMPC, DMPG, COL, DMPE	(G6PDH) G6P, NAD	Guinea pig serum	Serum	15 μg/ml	(170)
Phenytoin	DMPC, DMPG, COL, DMPE	(G6PDH) G6P, NAD	Guinea pig serum	Serum	5 μg/ml	(170)
Theophylline	PC, PG, COL, α-tocopherol	(G6PDH + G6P)	Guinea pig serum	Serum	2.5 μg/ml	(171)
Interferon-γ	DPPC, DPPG, COL, BX-DHPE	Carboxyfluorescin	triton-X	Serum	0.5 U/ml	(172)

PC, phosphatidylcholine; COL, cholesterol; POPC, L-α-palmityloleoylphosphatidylcholine; DMPC, dimyristoyl-phosphatidylcholine; DMPG, dimyristoylphosphatidylglycerol; PE, phosphatidylethanolamine; DMPE, dimyristoylphosphatidylethanolamine; PG, phosphatidylglycerol; DPPC, dipalmitylphosphatidylcholine; DPPG, dipalmitylphosphatidylglycerol; DPPE, dipalmitylphosphatidyl-ethanolamine; BX-DHPE, N-((6-biotinyl)amino)hexanoyl)-1,2-dihexadecanoyl-sn-glycero-3-phosphoethanolamine; FAD, flavin adenine dinucleotide; GOD, glucose oxidase; CL, chemiluminescence; ALP, alkaline phosphatase; PNPP, p-nitrophenylphosphate; G6PDH, glucose-6-phosphate dehydrogenase; G6P, glucose-6-phosphate; NAD, Nicotinamide adenosine dinnucleotide.

## IMMUNOASSAY FOR SIMULTANEOUS ANALYSIS OF MULTI-ANALYTES

Immunoassay methods can be applied for analysis of two or even more analytes in the same sample, employing different approaches. A dual-label assay was developed for the simultaneous analysis of thyroxine and triiodothyronine (173). Thyroxine was conjugated with alkaline phosphatase enzyme, and the triiodothyronine was conjugated with β-galactosidase enzyme. Competitive heterogeneous assays were performed for each hormone, in presence of each other. A double antibody precipitation technique was used for separating the immune complexes, followed by measuring the activity of the enzyme conjugates. The alkaline phosphatase conjugate was measured at 540 nm by the formation of phenolphthalein from its monophosphate salt. The β-galactosidase conjugate was measured at 420 nm by the formation of o-nitrophenol from o-nitrophenyl-β-galactoside substrate. The principles of this dual-label approach were used for the development of a similar system for the simultaneous analysis of phenytoin and phenobarbital in presence of each other (174).

A new heterogeneous FIA system was developed and automated for the simultaneous analysis of serum albumin and transferring (175). This system employed dual-fluorescent molecules as labels, and incorporated thiophilic gel solid phase reactor to separate antibody-bound and unbound molecules. Antibody elution was achieved by changes in ionic strength. Detection of the two separate fluorophores was achieved by high speed synchronous fluorescence scanning at two different wavelength intervals, one for each fluorophore.

A sensitive dual-label TRFIA was developed for the simultaneous analysis of methamphetamine and chloropromazine in serum (55). The assay is based on a novel coating strategy and enhanced lanthanide-based FIA technology. The coating strategy involved the coating of microwell plate wells with high concentrations of a mix Antibody Analyte Marker Signal Concture of anti-methamphetamine and anti-chloropromazine antibodies. Methamphetamine was labelled with Eu (III), and chloropromazine was labelled with Sm (III). In the assay, the labelled compounds were mixed with their non-labelled standard solutions or samples, and the mixture was then added to the wells coated with the antibodies. Methamphetamine was analyzed by measuring the fluorescence intensity of Eu (III) at 615 nm. Chloropromazine was analyzed by measuring the fluorescence intensity of Sm (III) at 643 nm. The assay showed rapid kinetics, and high sensitivities; the lower limits of detections were 1 and 10 ng/ml for methamphetamine and chloropromazine, respectively. A similar system was developed for the simultaneous analysis of thyroxine and thyroid-stimulating hormone in serum (176). The limits of detection for the assay were 4.1 nmol for thyroxine and 0.028 mIU/ml for thyroid-stimulating hormone with 20 μl sample volume.

A capillary-based FIA system was developed for the simultaneous determination of penicillin-G, ampicillin, amoxicillin, cloxacillin, cephapirin, and ceftiofur (177). The assay system consists of an assay cartridge containing 4 glass capillaries, a reagent tray with 4 wells of dried reagents, and a processor, which processes the assay, reads the fluorescent output, and finally reports the results. The assay system was specific, and sensitive; the limits of detection were 3.2, 2.9, 3.6, 7.4, 16.3, and 33.7 ng/ml for penicillin-G, ampicillin, amoxicillin, cloxacillin, cephapirin, and ceftiofur, respectively. The capacity of this system was subsequently increased to be able to the simultaneously analyze 9 compounds in the same samples (178). These compounds are tetracycines (tetracycline, oxytetracycline, chlorotetracycline, and doxycycline), cephalosporins (cephapirin, ceftiour and cefquinome), and b-lactam antibiotics (penicillin-G, ampicillin, and amoxicillin).

Microsphere-based competitive immunoassay was developed for the simultaneous analysis of digoxin and theophylline (179). This assay was performed using microspheres coated with both compounds, and antibodies labelled with horseradish peroxidase. Two flurogenic substrates were used to differentiate analytical signals from each compound. An epifluorescence microscope and a camera interfaced with a computer were used to measure the individual fluorescence signals. The measured signals were then related to the concentration of the compounds.

Carbonylmetalloimmunoassay method was developed for the simultaneous analysis of carbamazepine, phenobarbital, and diphenylhydantoin (180, 181). This assay used various transition element-carbonyl complexes as labels followed by the sensitive quantitation of these complexes by Fourier transform infrared spectroscopy. The measurements utilized the particular spectral features of the carbonyl complexes, which show very strong absorption bands in the 1800-2200 cm-1 region. The signals were individually assignable and the intensities were used for quantitation.

## ADVANCES IN IMMUNOASSAYS FOR PHARMACEUTICAL ANALYSIS

Advances in immunoassay continue in all areas of the technology; preparation of the unique reagents, analysis of new categories of compounds, and improvement in the methodology and instrumentation:

### Advances in Preparation of the Immunoanalytical Rreagents

**Figure 4:**
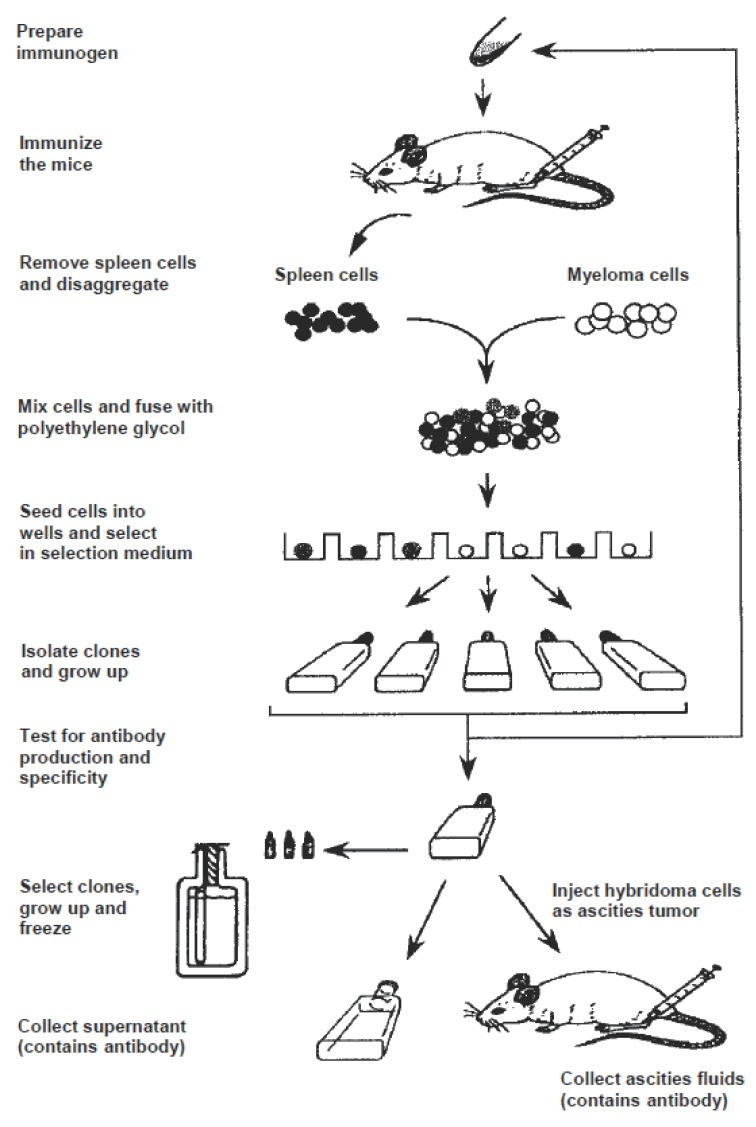
Schematic diagram for production of monoclonal antibodies.

Immunoassay methods, particularily the monoclonal antibody-based assays, are of potential advantages in parmaceutical analysis because of their high-throughput and high-sensitivity for the analysis in biological samples. However, the protocol for preparation of a specific monoclonal antibody for small drug molecules (Figure 4) is difficult because the low incidence of positive hybridomas from which selection of the most specific one is performed. Furthermore, the production time is excessive (typically 3-9 months). Recent studies were performed to solve these problems. Molecular modeling of the hapten structure was emerged as a useful tool in predicting the immunogenicity of the immunogen, and subsequently the specificity of the generated antibody. By this technique, it was possible to speculate if the antigenic epitope of the hapten molecule is projected and exposed to the immune system, or enveloped into the molecular structure, and thus losses its immunogenicity (182). This approach was used for synthesis of a sulphacetamide-protein immunogen aimed to the generation of broad specificity antibodies for sulphonamide drugs (183).

A novel immunization technique was developed for the rapid production of monoclonal antibodies (184). This technique employed a repetitive, multiple sites, and short regime (8-13 day) immunization. This immunization was followed by fusion of popiteal and bracial lymph node cells from the immunized mouse with myeloma cells. The antibody production time, including immunizations, fusion, screening and cloning takes ~30 days to be completed. This technique was used for generation of monoclonal antibody specific to ranitidine; the generated antibody was used in the development of an immunoassay method for ranitidine (27). An efficient shorter immunization regime was developed by Darwish *et al*. (26). This technique employed only a single immunization of rats, rather than mice. Two weeks later, cell fusion is performed between the medial iliac lymph nodes and rat myeloma cells. By this technique, high number of positive hybridomas was obtained, which offered the chance for selection of more specific hybridoma cells. This technique was employed for generating of highly specific monoclonal antibody for 2`-deoxycytidine; marker for the prognosis of breast cancer to chemotherapy (185). The generated antibody was subsequenly used for development of specific immunoassay method for this marker in plasma (186). The use of only single injection in this technique saves not only the time and effort given to the purification of the immunogens (usually by time-consuming chromatographic procedures), but also the amount of the immunogen which is often very valuable or difficult to obtain in an amount enough for multiple injections, or difficult to purify.

### Advances to Involve New Categories of Compounds

**Figure 5:**
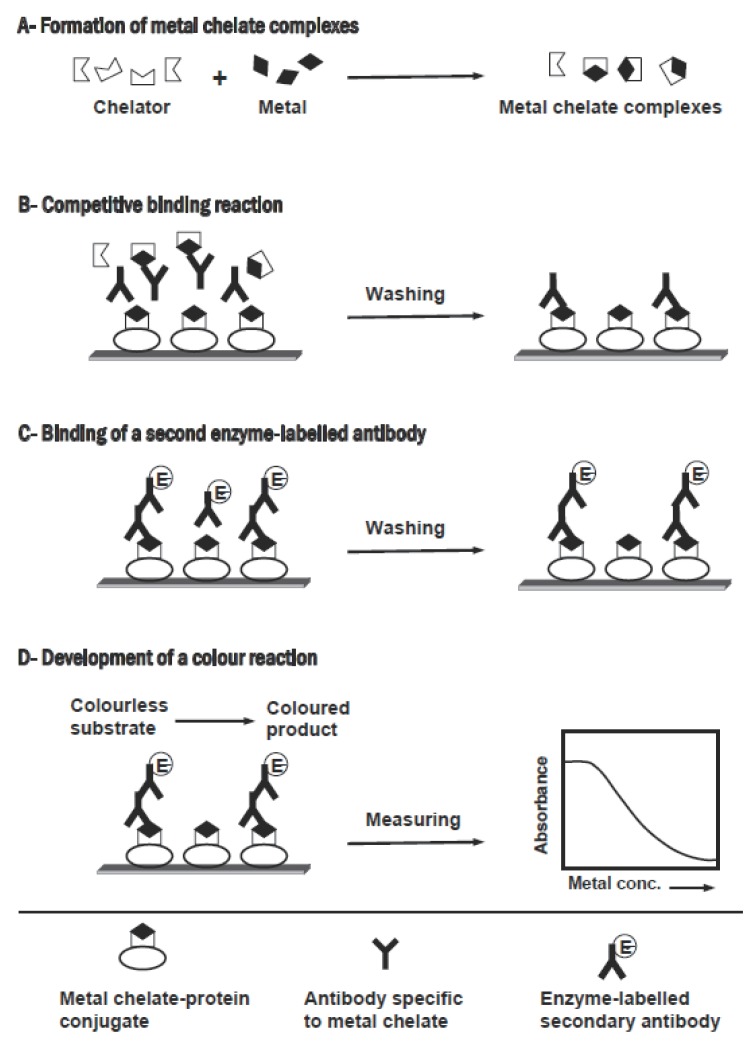
Schematic diagram of the competitive immunoassay for metal ions.

Most of immunoassay methods applied in pharmaceutical analysis are directed toward organic compounds, however this technique is theoretically applicable to any analyte, even inorganic metal ions, if a suitable antibody can be generated. The ability of generating monoclonal antibodies that recognize metal ions has been demonstrated (187-189). The immunogens were prepared by chelating the metal ions with a bifunctional chelator (e.g. p-isothiocyanate-benzyl EDTA), followed by coupling the metal-chelator complex to a carrier protein via the isothiocyanate group of the chelator. The screening assay was designed to select hybridomas that secrete antibodies that show higher affinity to the metal-chelator complex, rather than to the metal-free chelator. By this approach, specific monoclonal antibodies were generated for uranium (182), cadmium (187), lead (188), cobalt (189), and mercuric (191, 191). These antibodies were used in the development of competitive immunoassays for the accurate measurement of these metal ions in water samples (190-195) as well as in human serum (196). The basic protocol for the competitive immunoassay for metal ions is illustrated in Figure 5. Briefly, the sample containing metal ions is treated with an excess of metal-free chelator to form metal chelate complexes. The resulting solution, which contains the excess chelator and the metal chelate complexes, is mixed with the antibody in microwell plates containing immobilized metal chelate-protein conjugate. The soluble metal chelate complexes compete with the immobilized metal chelate-protein conjugate for the binding sites of a specific antibody. After attaining the equilibrium, the unbound reagents are removed by washing, and an enzyme labelled secondary antibody is added. The unbound secondary antibody is reomved by a second wash step. The signal is generated by addition of a chromogenic enzyme substrate. The measured signal is then related to the concentration of metal ions present in the original sample. These assays were highly sensitive, remarkably quick, easily performed, reasonably portable to the analysis site, require minimum sample pretreatment, and inexpensive.

More recently, FPFIA methods were developed for lead (II) and cadmium (II) (197). The antibodies were raised against protein conjugates of the metal ionsplexes of polyaminopolycarboxylate chelating agent. Fluorophore-labelled analogues of the metal chelate complexes were used as tracer in performing the competitive assay. This method was very sensitive; the limits of detection were 20, and 100 ppt for lead (II) and cadmium (II), respectively.

Although immunoassays for metal ions are still in their infancy, very promising results have been obtained. Recent advances in the genetic manipulation of recombinant single-chain antibodies provided a new opportunity to optimize the binding properties of the parent antibody to provide new highly specific recombinant reagents (198). The new immunoassays for heavy metals are considered as a promising alternative approach for the existing technologies for analysis of metal ions (atomic absorption spectroscopy, inductively coupled plasma emission spectroscopy, etc.), which are expensive and the analysis needs excessive pretreatment of the sample.

## ADVANCES IN METHODOLOGY AND INSTRUMENTAION

### Cloned Enzyme Donor Immunoassay

**Figure 6:**
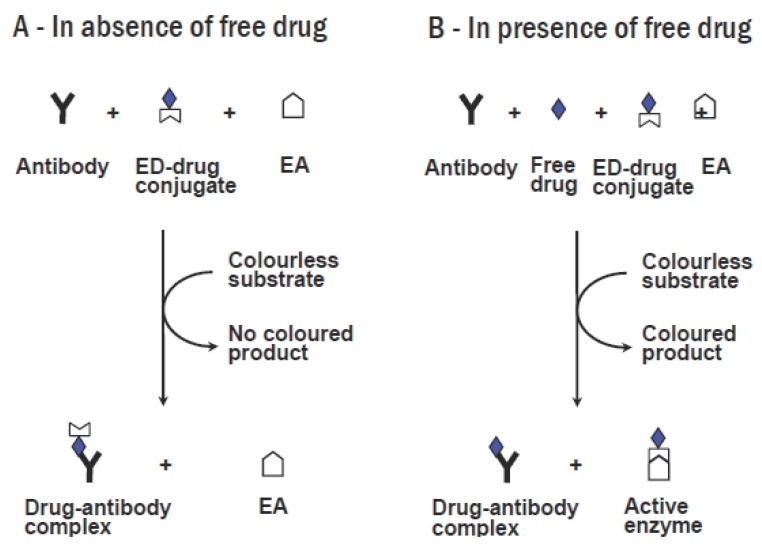
Principles of the cloned enzyme donor immunoassay. (A) In the absence of free drug, formation of a complete tetrameric enzyme is inhibited, and no coloured product is generated after addition of substrate to the reaction mixture. (B) In presence of free drug, it competes with the enzyme donor (ED)-drug conjugate for anti-drug antibody binding sites. Complete active enzyme molecules are formed, which converts the colourless substrate into coloured product in proportional to the drug concentration. EA, enzyme acceptor.

Cloned enzyme donor immunoassay (CEDIA) methodology is a novel approach which uses the DNA technology to produce homogenous enzyme immunoassays for drugs. The principle of this method is illustrated in Figure 6. Enzyme donor units combine with enzyme acceptor units to form a complete and fully active tetrameric enzyme molecule, which reacts with a colourless substrate to produce coloured product. An enzyme donor-drug conjugate is prepared by linking the drug molecule to the enzyme donor fragment. Competitive binding reaction results in the formation of active enzyme and consequently coloured product, which is directly proportional to the concentration of the drug present. This assay is rapid and has high throughput. Successful cloned enzyme donor immunoassay methods were developed for analysis of amphetamine, methamphetamine (199, 200), barbiturates, opiates, phencycline (201), phenytoin (202), and benzodiazepines (203-205). The CEDIA methods were validated, in terms of the sensitivity and precision, in reference to gas chromatography- mass spectrometry, and their validity was proved for application in routine drug screening (206-208).

### Flow-Injection Immunoassay

**Figure 7:**
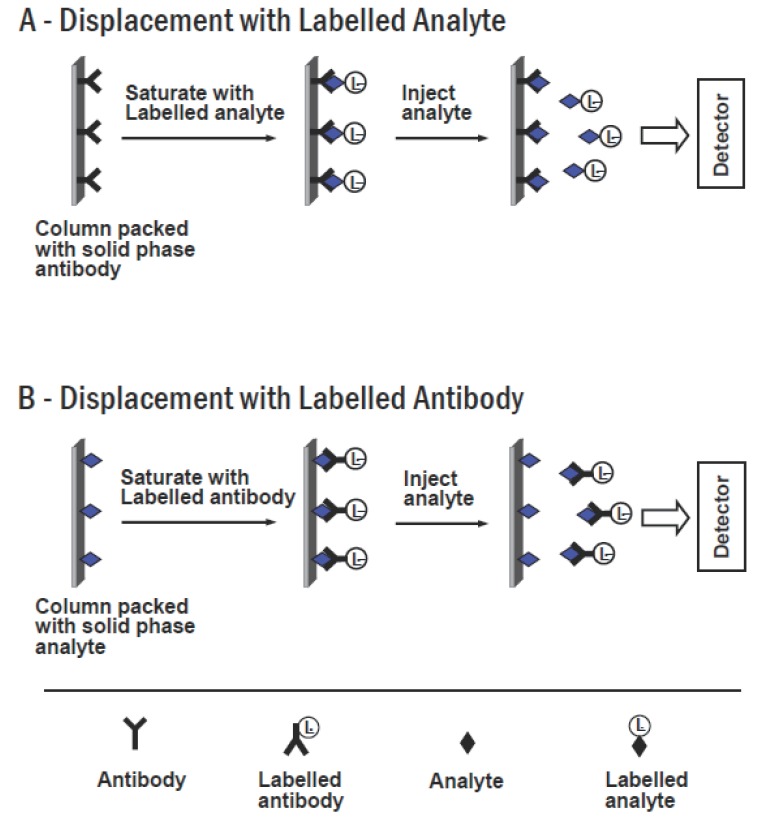
Schematic diagram of displacement flow-injection immunoassay.

Flow-injection immunoassay (FIIA) methods were recently introduced to enhance the efficiency of immunochemical reaction, as well as to increase the performance of the analysis. Most of the developed FIIA systems are based on a principle of displacement (Figure 7). In this case, a column packed with immobilized solid phase antibodies is incorporated into the system. The column is saturated with a solution containing labelled analyte. Injection of the sample (contains the free unlabelled analyte) into the column results in displacement of the labelled analyte (due to the affinity of antibody for labelled analyte is usually significantly lower than its affinity for unlabelled free analyte). The displaced labelled analyte is eluted and detected at the outlet of the column. The generated signal is directly proportional to the concentration of the analyte in the sample (Figure 7A). A similar system was constructed using a column packed with immobilized analyte, and presaturated with labelled antibody. The injection of the sample into this system results in displacement of the analyte-labelled antibody complexes, which is the detected at the outlet of the column (Figure 7B).

**Figure 8:**
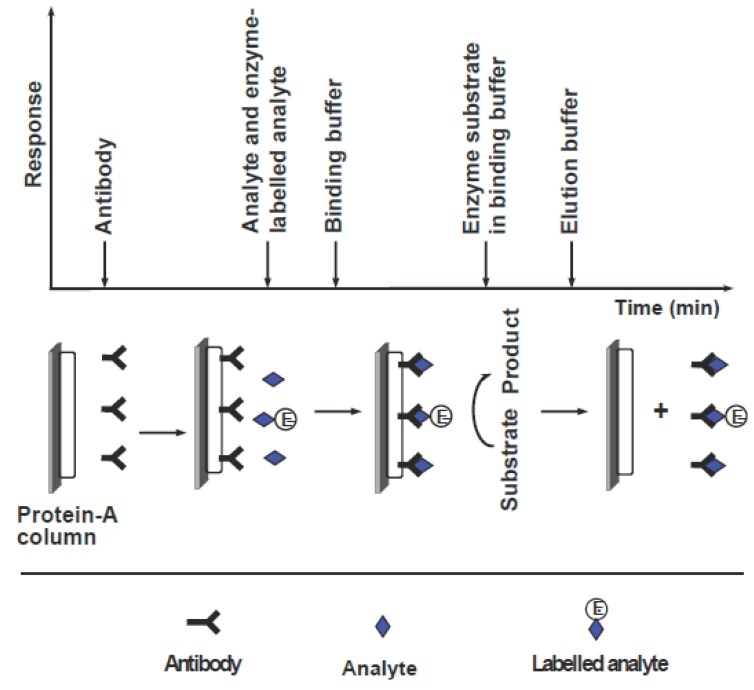
Schematic diagram for a reaction cycle of the flow-injection enzyme immunoassay.

FIIA systems based on displacement were developed for analysis of gentamicin (209), phenobarbital (210), and digoxigenin (211). The limits of detections of these flow-injection systems were in the range of 10-100 nM. The prototype of a flow-injection based instrument has been constructed by United States Drug Testing to screen urine for the presence of trace amounts of drugs of abuse (212). For analysis by displacement-based FIIA, definite conditions such as column, flow rate, and buffer solution should be optimized for each particular analyte, and should be met in the analysis apparatus. To overcome this diasadvantage, an alternative system was developed for theophylline (213). The general assay process involved in this system is shown in Figure 8. This system consists of a column containing immobilized protein-A, which possess the property of capturing most antibodies with high affinity. The preliminary step in the analysis is a saturation of the column with anti-theophylline antibody. The analysis is conducted by injection of a mixture of free theophylline and theophylline labelled with alkaline phosphatase enzyme. The labelled and free theophylline compete to interact with the antibodies bound to protein-A. The amount of labelled theophylline bound to antibodies is inversely proportional to the concentration of free theophylline. This amount is determined by injecting p-aminophenylphosphate substrate and amperometric detection of the produced p-aminophenol. After the assay procedure, the column is regenerated by removing complexes by acid elution through the column. Immobilized protein-A is then saturated again with new antibodies, and the assay is repeated. This system is time consuming since four steps are involved for each measurement, i.e. incubation of reagents, separation on the column, injection of substrate, and finally regeneration of the protein-A column. The applications of this approach of protein-A column regeneration was applied in the analysis of various compounds (214).

**Figure 9:**
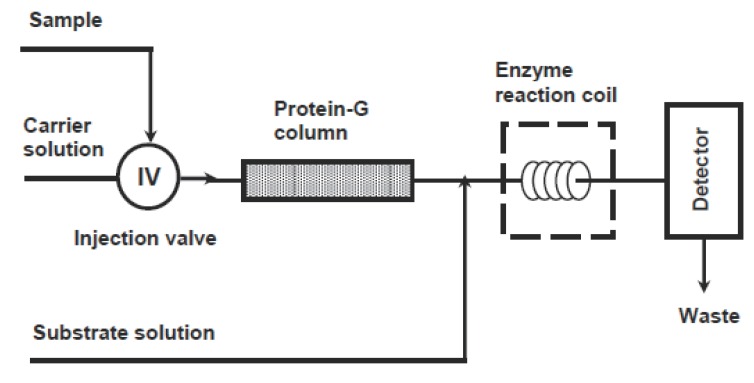
Schematic diagram for the continuous-flow enzyme immunoassay for determination of aminoglycosides.

An improved flow system has been developed by Darwish (215) for analysis of aminoglycosides (e.g. tobramycin) in human serum. A schematic diagram for this assay system is shown in Figure 9. This system is based on off-line competitive reaction between tobramycin and tobramycin labelled with β-galactosidase enzyme for the anti-tobramycin antibody. After the equilibrium was reached, the sample was introduced into the flow system. The antibody complexes of both free tobramycin and labelled tobramycin were trapped in a protein G column while the unbound labelled tobramycin was eluted and monitored by colorimetric detection down-stream after reaction with chlorophenolic red-β-D-galactopyranoside as a substrate. The signal was directly proportional to the concentration of tobramycin in the original sample. Protein-G column used in this system showed a good stability and capacity, thus allowing analysis of large number of samples before the need for regeneration of the column (216). Furthermore, the continuous detection of the eluting free fraction of the labelled tobramycin, rather than the bound fraction, allowed a higher sample throughput.

### Capillary Electrophoresis Immunoassay

Capillary electrophoresis immunoassay (CEIA) has been recently introduced as a sensitive analytical technique, particularly when combined with a sensitive detection method. This technique is a combined use of the principles of the capillary electrophoresis separation and the heterogeneous immunoassay. In this technique, the antibody is attached covalently to the modified interior surface of a microcapillary, which is used as a solid phase immunoreactor. The detection is based on the reaction process of the immunoassay. The analyte and labelled analyte (tracers) are mixed and the mixture is then injected into the system. Tracers are detected with on-column detector. The concentration dependent peaks produced serve as the basis for quantitation. These principles were utilized in the development of an assay system for determination of phenobarbital in serum (217). Alkaline phosphatase labelled phenobarbital was used as tracer, and p-aminophenyl phosphate was used as a substrate. The detection was carried out amperometrically for the produced p-aminophenol. The capillary electrophoresis with competitive assay design was capable to achieve detection limits in the picomole levels (217, 218) for less than 5 ml sample volume.

A CEIA based on homogeneous fluorescence polarization was developed. The imunoanalytical reagents (analyte, fluorescein-labelled analyte, and antibody) are mixed off-line, and the mixture is then injected into the electrophoresis capillary. The fluorescein-labelled analyte and the antibody-labelled analyte complexes are separated under applied potential, and detected by on-column laser-induced fluorescence detector. This system was applied to the analysis of various drugs in biological fluids, e.g. methotrexate (16), methamphetamine (219, 220), theophylline, ethosuximide, paracetamol, salicylate, quinidine (221), hirudin (222), codeine, dihydrocodeine, and their glucuronides (223).

A sensitive CEIA method with electrochemical detection was developed for the analysis of thyroxine in human serum (44). The method is a combined use of capillary electrophoresis separation and homogeneous enzyme immunoassay principles. The assay was performed in competitive configuration between sample thyroxine and horseradish peroxidase-labelled thyroxine for anti-thyroxine antibody. The free enzyme-labelled thyroxine and the antibody-bound labelled thyroxine complexes were separated by capillary electrophoresis in a separation capillary. Then, they catalyzed the oxidation of tetramethylbenzidine substrate with hydrogen peroxide in a reaction capillary connected to the system after the separation capillary. The reaction product of the substrate was amperometrically determined using an electrode at the outlet of the reaction capillary. The assay was very sensitive; the limit of detection was 23.2 attomole. A similar system was developed for cortisol, with limit of detection of 7.8 attomole (224). The same principle was used in designing a sensitive assay for tumor markers (225).

The CEIA with fluorescence detection was employed for the simultaneous analysis of urinary methadone, opiates, benzoyl-ecgonine (cocaine metabolite), and amphetamine (226). After incubation of the sample with the imunoreactants, a small aliquot of the mixture is applied onto a fused silica capillary and the unbound fluorescein labelled drugs were monitored by capillary electrophoresis with on-column laser induced fluorescence detector. This multi-analyte system was also applied for analysis of digoxin and gentamicin (227).

### Immunosensors

Immunosensors represent the most technological progress in the field of immuoassay development (228-233). These sensors are analytical devices composed of an immunochemical recognition element directly interfaced to a signal transducer, which together relate the concentration of an analyte to a measurable response. Wide range of signal transducers can be used, e.g. optical, electrochemical, and piezoelectric transducers. The principles underlying immunosensors technology, and their analytical applications are the subject of many reviews (234-237). Immunosensors were widely applied in the analysis of many compounds of pharmaceutical importance (238-245); examples are given in Table 9. These sensors usually employ either reusable/regenerable or disposable assay format. Sensors using disposable formats are potential for multi-analyte analysis, format versatility, system cost, the assay time, sensitivity, and reproducibility. In contrast to the disposable formats, the multi-use immunosensors (which can recharged or regenerated) offer certain advantages, particularily for use as detector for chromatographic and flow injection systems. For example, continous flow and electrochemical immunosensors developed for analysis of cephalexin (246), paclitaxel (247), progesterone (248), and thyroid hormones (249).

**Table 9:** Immunosensors for some Compounds of Pharmaceutical Importance

Immunosensor type/compound	Sensitivity	Ref.

Electrochemical sensor		
Biotin	0.01 μg/ml	(238)
Cocaine	0.1 μmol	(239)
Cortisol	0.1 μmol	(240)
Lutinizing hormone	1 ng/ml	(241)
Piezoelectric sensor		
Cocaine	229 nmol	(242)
Cortisol	36 ng/ml	(243)
Optical sensor		
Theophylline	150 μmol	(244)
Benzoylecgonine	0.1 μg/ml	(245)

Although many immunosensors were developed for analysis of wide range of drugs and hormones. However, it was not possible to perform the analysis in complex biological fluid (e.g. serum), in a one-step procedure, due to the interference resulting from the non-specific binding to the sensor surface. To control this interference, an optical chip interferometer has been developed (250). This interferometer configuration employed a reference sensing region that can be functionalized separately from the measuring sensing region, and additional reduction in signal drift in serum was achieved by controlling the surface chemistry of the optical chip. This functionalized procedure was utilized in the development of one-step assay for human chorionic gonadotropin in human serum with a detection limit of 0.1 ng/ml for a 35 min assay.

## CONCLUSIONS

Immunoassay methods are bioanalytical methods in which quantitation of analyte depends on its reaction with specific antibody. The response signal is generated from a label attached to either the analyte or antibody. The property of highly specific recognition of analytes by antibodies leads to the high selectivity of these assays. The extreme affinity of analyte-antibody interaction results in greater sensitivity of immunoassay methods. The instrumentation technology leads to automation of immunoassays and consequently increasing their throughput. Immunoassay methods are capable of quantifying wide variety of compounds such as low molecular weight drugs, macromolecular biomolecules, metabolites, and/or biomarkers which indicate disease diagnosis or prognosis. Therefore, these methods found wide applicability in many important areas of pharmaceutical analysis such as diagnosis of diseases, therapeutic drug monitoring, clinical pharmacokinetic and bioequivalence studies in drug discovery and pharmaceutical industries.

Inspite of the numerous advantages of immunoassays, they have some limitations. Immunoassays depend mainly on reaction between analyte and a biological antibody, they may have more inherent imprecision than other methods employed in pharmaceutical analysis (e.g. chromatography). The specificity of immunoassays depend mainly on the antibody directed to the analyte, however some immunoassays are not highly selective, and they may respond to a group of compounds (e.g. aminoglycosides, pesticides,...etc.) rather than individual compounds. Moreover, lack of specificity may be observed due to non-specific binding of the antibody to matrix component(s). In these circumstances, care must be taken to ensure the absence of interfering- substance in the analyte sample and/or matrix.

Recently, a marked improvement was achieved in the field of immunoassay development for the purposes of pharmaceutical analysis. This improvement involved the preparation of the unique immunoanalytical reagents, analysis of new categories of compounds, methodology, and instrumentation. The most important examples in this field are the development of continuous-flow immunoassays and immunosensors. These technologies resulted in improvement of the analysis performance by increasing the sensitivity, decreasing the analysis time, simplification of the assay procedure, automation of the method, and miniaturization of the analytical equipment.
